# Naloxone Availability, Testing Drugs for Potency, and Solitary Use: Unpacking the Determinants of Overdose Prevention Behaviors

**DOI:** 10.1080/10826084.2025.2600637

**Published:** 2025-12-17

**Authors:** Carl. A. Latkin, Lauren Dayton, Haley Bonneau, Melissa A. Davey-Rothwell, Leane Santos-Silva, Grace Yi, Oluwaseun Falade-Nwulia

**Affiliations:** a Department of Health, Behavior and Society, Johns Hopkins Bloomberg School of Public Health, Baltimore, Maryland, USA; b School of Education, Johns Hopkins University, Baltimore, Maryland, USA; c Division of Infectious Diseases, Johns Hopkins School of Medicine, Baltimore, Maryland, USA

**Keywords:** Overdose, opioids, naloxone, prevention, potency, gender, race

## Abstract

**Background:**

Fatal and nonfatal opioid overdoses remain a pressing public health challenge. However, engagement in drug overdose prevention and response behaviors may vary across demographic and social contexts.

**Objectives:**

This study examines individual and social determinants of these behaviors among people who use opioids (PWUO), leveraging data from the OASIS study in Baltimore, Maryland (*N* = 783). Ordered logistic regression models assessed factors associated with three key behaviors: testing-dosing to assess drug potency, naloxone availability while using with others, and solitary drug use.

**Results:**

The three key overdose prevention behaviors were not strongly correlated with one another. Racial disparities emerged, with Black participants more likely to engage in test-dosing compared to White participants. Gender differences were also notable, with women less likely to use with others who have naloxone available when using drugs. Social network factors played a key role; having a “running buddy” was strongly protective against solitary drug use.

**Conclusions:**

These findings underscore the importance of tailored harm reduction interventions that address racial and gender disparities, enhance social networks, manage withdrawal, and enhance naloxone availability. Integrating harm reduction skill training into peer-driven naloxone distribution and overdose prevention programs, training non-drug-using network members, and addressing structural barriers may enhance overdose prevention strategies.

## Introduction

The opioid dependence and overdose crisis, characterized by high rates of fatal and nonfatal overdoses that devastate individuals, families, and communities, represents one of the most significant public health challenges in North America.

Among people who use opioids (PWUO), specific behaviors play a critical role in mitigating the risk of fatal overdose. These behaviors include testing drugs for potency, ensuring the presence of others during drug use, and having naloxone readily available to reverse overdoses (Taha et al., 2019). Given the diversity of fatal overdose prevention behaviors that PWUO can practice, there is a need to understand how these prevention behaviors are related to each other and how individual and social factors influence them.

In the United States, provisional data indicate there were an estimated 107,543 drug overdose deaths during 2023. (Ahmad et al., 2025). Historically, Baltimore has had one of the highest rates of fatal drug overdoses in the U.S. In 2023, Baltimore City recorded 1,043 intoxication deaths, 88% involved fentanyl, and 8,094 intoxication deaths over a 10-year period. The age adjusted mortality rate in 2023 was 121.3 per 100,000 population (Maryland Department of Health, 2025). Nonfatal overdoses also contribute to the crisis, often resulting in long-term physical and psychological health consequences and straining healthcare systems (Hawk et al., 2015). These rates and the physical and emotional toll of drug overdose highlight the urgency of implementing harm reduction measures and addressing barriers to their adoption.

Naloxone is an essential component of harm reduction. Despite its life-saving potential, barriers impede its accessibility and utilization (Dayton et al. 2021). Geographic disparities in distribution, cost, stigma, and insufficient training undermine the effectiveness of naloxone programs (Miller et al., 2022). Misperceptions, such as fears that naloxone encourages riskier drug use, and legal concerns, such as fears of arrest despite Good Samaritan laws, further deter individuals from carrying or using naloxone (McDonald et al., 2017). Efforts to expand access through pharmacies, community organizations, and public health campaigns have been essential to overcoming these challenges and normalizing naloxone use (Bailey & Wermeling, 2014).

Research has demonstrated that social network influences can either facilitate or hinder engagement in harm reduction practices (Boyd et al., 2017; Green et al., 2009; Gupta et al., 2014). Social networks shape individuals’ attitudes and practices regarding harm reduction. For instance, individuals who have larger drug networks have been found to be more likely to overdose (Latkin et al., 2004) and are more likely to have responded to multiple overdoses (Marks & Wagner, 2022). People who inject drugs (PWID) are more likely to participate in naloxone training if their social networks include non-injecting drug users or individuals who have personal connections to overdose victims (Wagner et al., 2013).

Social norms also influence drug use behaviors. A systematic review of opioid use in rural communities suggested that entrenched stigma and social norms often prevent individuals from seeking overdose prevention training or using naloxone (Bolinski et al., 2019). Social norms governing drug use behaviors can play a significant role in shaping overdose risk. For example, people who use heroin in environments where smoking is more common than injecting are less likely to transition to injection drug use, which carries a higher risk of overdose (Harris et al., 2020). Mutual aid within drug-using networks and communities plays an important role in overdose prevention. Research indicates that informal peer-led harm reduction strategies, such as sharing naloxone kits, training peers on how to recognize overdoses, and encouraging safer drug use practices, contribute to a culture of overdose prevention (Mercer et al., 2021).

Individual factors also have a critical role in overdose prevention. The severity of withdrawal symptoms, for example, can drive individuals to prioritize immediate relief over safer practices such as waiting for others to be present or testing substances (Jha & Madison, 2012). Avoidance and treatment of withdrawal symptoms may lead to quickly ingesting drugs and, hence, compromised overdose prevention behaviors (Bluthenthal et al., 2020; Elliott et al., 2024). Drug purchasing patterns may also influence harm reduction practices by buying from the same dealers, routinizing behaviors and increasing confidence in drug quality. Having a drug “running buddy” (someone that PWUO uses drugs with frequently) may also influence harm reduction behaviors. Having a running buddy may heighten concern about peer overdose, motivate PWUO to always carry naloxone, and reduce using drugs alone.

This study seeks to investigate the interplay between social and individual factors in shaping overdose prevention behaviors. Specifically, we examined whether overdose prevention and care behaviors of testing drugs for potency, ensuring the presence of others during drug use, and having naloxone readily available to reverse overdoses are correlated. We then assessed the social and individual factors linked to these behaviors. By identifying these factors, the study aims to inform the development of targeted, evidence-based interventions that address the multifaceted nature of the opioid crisis.

## Methods

Participants were from the OASIS study, which assesses geospatial and social factors linked to drug overdoses. Between December 7, 2022, and January 26, 2025, 792 individuals enrolled in the study. Study participants were recruited through community outreach and word-of-mouth in areas with high drug use in Baltimore, Maryland. Enrollment criteria included self-reported illicit opioid use in the prior 30 days, age of 18 years or older, and living in the Baltimore Metropolitan area. All study protocols were approved by the Bloomberg School of Public Health IRB. Participants were paid USD 40 for the visits. The final analytic sample included 783 individuals with complete data.

### Measures

The three study outcomes assessing overdose prevention and care behaviors were (1) frequency of test dosing measured by response to the question “How often do you use a small dose first to see how strong your drugs are,” (2) frequency of solitary drug use measured by response to the question “How often do you use drugs alone?,” (3) frequency of availability of naloxone to provide the participant during drug use measured by response to the question “When you are using with others, how often do they have Narcan with them?”. Response options included 1-Always, 2-Often, 3-Sometimes, 4-Rarely, and 5-Never. These response options were included in analyses as an ordinal outcome.

To assess comfort in using naloxone, participants were asked, “How comfortable are you using nasal naloxone on someone else?”(response options: 1-Very comfortable, 2-Somewhat comfortable, 3- Somewhat uncomfortable, 4- Very uncomfortable).

## Individual-level factors

Socio-demographic characteristics, including age, race (categorized as Black, White, or Other), level of education, sex at birth, and experience of homelessness in the prior six months, were self-reported in study surveys. The response categories for race included “African American/Black,” “White,” “Asian,” “Other,” and “Refuse to answer.” Due to few responses in multiple categories, “Asian,” “Other,” and “Refuse to answer” were collapsed into a single category. Sex was assessed as “male” and “female.” Educational attainment was categorized into four groups: (1) Less than 12th grade, (2) Grade 12 or GED, (3) Some college or Associate’s degree, (4) Bachelor’s degree or greater.

Frequency of drug (heroin, fentanyl, and crack cocaine) use was assessed for the prior three months. Number of drug use locations was assessed based on response to the question, “Thinking about the places that you use drugs, how many different places have you used in the past month?” The number of places participants used in the past 30 days was measured continuously and condensed to values of one through ten, with values greater than ten categorized as eleven.

To assess perceptions of the impact of withdrawal symptoms on overdose prevention behaviors, we included the question, “Sometimes when I feel sick or in withdrawal from not having my fix, I don’t think about overdose prevention.” (response options: Strongly Agree, Agree, Neither Agree nor Disagree, Disagree, Strongly Disagree). Frequency of drug purchases from the same individual was assessed by response to the question, “How often do you buy drugs from the same people?”

### Social network and norms

Social norm questions assessed descriptive norms related to overdose prevention behaviors. These included, “How often do people that you use with use a small dose first to see how strong their drugs are?” (response options: Always, Often, Sometimes, Rarely, Never), and “Thinking of the people you know who use drugs, how many do you think have Narcan?” (response options: None, Some, Half, Most, All). Stigma related to carrying naloxone was assessed with the questions, “If I carry Narcan, I am doing something that is helping the community” and “If I carry Narcan, I feel negatively judged by people in my community (response options: Strongly Agree, Agree, Neither Agree nor Disagree, Disagree, Strongly disagree). Having stable drug use partners was assessed with the question, “Do you have a running buddy(ies)? (A running buddy is someone that you hustle with, go in together with, and/or often use with)” (yes/no).

### Analyses

Descriptive statistics and Kendal Tau correlations were first conducted among the three overdose behaviors. Bivariate and multivariate ordered logistic regression models were used to evaluate differences between respondents based on their frequency of engagement in the following behaviors: (1) “How often do you use a small dose first to see how strong your drugs are?,” (2) “How often do you use drugs alone?” and (3) “When you are using with others, how often do they have Narcan with them?” After reviewing the distributions, the response categories for “How often do you use a small dose first to see how strong your drugs are?” were condensed into (1) Always/Often, (2) Sometimes, and (3) Rarely/Never. The response categories for “How often do you use drugs alone?” and “When you are using with others, how often do they have Narcan with them?” were collapsed into (1) Always/Often, (2) Sometimes, and (3) Rarely/Never and reversed coded for the ordered logistic regression model.

Sociodemographic variables were included in the multivariate ordered logistic regression models regardless of their statistical significance. These sociodemographic variables were race, age, sex, and educational attainment. Non-sociodemographic variables were included in the multivariate models if p< =0.15 in the bivariate models. The statistical software Stata 17 was used for the analyses.

## Results

The study included 783 participants, with a majority identifying as Black (71.5%), followed by White (22.7%) (Table 1). Regarding sex assigned at birth, 61.2% were male, and 38.8% were female. The mean age in years was 49.8 (SD = 10.9), and the median was 52. The educational background of participants varied, with 29.4% having completed Grade 11 or less, 45.1% earning a high school diploma or GED, 22.7% attending some college or earning an associate’s/technical degree, and 2.8% obtaining a bachelor’s degree or higher. Experiencing homelessness in the past six months was reported by 44.8%. A large proportion of participants were unemployed (43.0%) or disabled/unable to work (42.5%).

Most (78.5%) respondents reported daily or almost daily use of fentanyl or heroin, and 43% reported daily or almost daily crack use. Regarding fatal overdose prevention behaviors, when asked about using a small test dose before consuming drugs, 41.9% reported “always” doing so, 37.3% reported “often” or “sometimes,” and 20.8% reported “rarely” or “never” using this harm reduction strategy (Table 1). Regarding drug use alone, 48.0% reported “always” or “often” using alone, 29.0% reported “sometimes,” and 23.0% reported “rarely” or “never.” In response to the question, when you are using with others, how often do they have Narcan with them? 23.37% reporting “always” or “often” having Narcan, 35.51% reporting “sometimes,” and 41.1% reporting “rarely” or “never” having it on hand.

Regarding drug acquisition habits, most participants reported frequenting the same drug supplier, with 34.6% “always” buying from the same person and 49.4% “often” doing so.

The majority (60.9%) of participants reported having a running buddy. Participants were asked to estimate how many of the people they know who use drugs have Narcan (Table 2). Over half (57.3%) of respondents reported that some of the people they know have Narcan, while 15.3% reported “most” and 5.5% reported “all” having Narcan. However, 12.4% reported that none of the people in their network had Narcan. Participants were questioned about how often their peers use a small test dose before consuming a full dose. The responses were distributed across categories: 8.3% reported “always,” 13.4% “often,” 25.7% “sometimes,” 24.3% “rarely,” and 28.4% “never.”

Most participants (67.3%) reported feeling “very comfortable” administering intranasal Narcan. However, a smaller percentage expressed discomfort, with 14.1% “somewhat uncomfortable” or “very uncomfortable.” The vast majority (94.2%) of participants agreed or strongly agreed that carrying naloxone was helping the community, and approximately two-thirds (67.4%) agreed or strongly agreed that when they had withdrawal symptoms, they were less likely to think about overdose prevention (Table 3).

The correlations among the three prevention behaviors were small, with a positive correlation between the frequency of having naloxone available when using and the frequency of testing drugs for potency (0.04, NS), a negative correlation between frequency of having naloxone available when using and frequency of using alone (−0.07, *p* < 0.01), and the correlation between frequency of testing drugs for potency and frequency of using alone was close to zero (−0.01, NS).

### Solitary drug use

Compared to Black participants, White participants were significantly less likely to use drugs alone (aOR = 0.57, 95% CI [0.39, 0.84]), (Table 4). Female participants had a lower unadjusted odds of frequency of using drugs alone (OR = 0.76, 95% CI [0.58, 0.99]), but this association was no longer significant in the multivariate model.

Having a running buddy was strongly associated with lower odds of frequency of using drugs alone (aOR = 0.48, 95% CI [0.36, 0.64]). The great number of places where participants used drugs was also associated with more frequent solitary drug use (aOR = 1.08, 95% CI [1.04, 1.13]). Greater comfort with using Narcan on others was associated with lower odds of solitary drug use (aOR = 0.82, 95% CI [0.70, 0.97]). Those reporting a larger proportion of people in their networks who used a small test dose first to check drug potency were significantly less likely to use drugs alone (aOR = 0.87, 95% CI [0.78, 0.97]).

### Naloxone availability while using

There were 124 participants removed since these participants did not report using drugs with others; hence, this outcome included 659 participants. Older age was associated with a lower likelihood of having naloxone available (aOR = 0.98, 95% CI [0.96, 0.99]) (Table 5). Females were significantly less likely than males to use drugs with others who have naloxone (aOR = 0.72, 95% CI [0.52, 1.00]). Knowing more people who have naloxone was associated with a significantly higher likelihood of naloxone availability (aOR = 2.25, 95% CI [1.91, 2.64]). Having a running buddy was also positively associated with naloxone availability (aOR = 1.74, 95% CI [1.25, 2.42]). Also, feeling negatively judged for carrying Narcan was positively associated with naloxone availability (aOR = 1.15, 95% CI [1.01, 1.31]).

### Test-dosing

Compared to Black participants, White participants were significantly less likely to use a small test dose before consuming a full dose (aOR = 0.54, 95% CI [0.37, 0.78]) (Table 6). Female participants were significantly more likely to use a small test dose compared to males (aOR = 1.42, 95% CI [1.06, 1.89]). The frequency of buying drugs from the same person was positively associated with test dosing (aOR = 1.31, 95% CI [1.09, 1.57]). If carrying naloxone was more strongly endorsed as helping the community, participants were significantly more likely to use a test dose (aOR = 1.34, 95% CI [1.11, 1.63]). Comfort using nasal naloxone on someone else was significantly associated with increased odds of using a small test dose (aOR = 1.20, 95% CI [1.02, 1.40]), and frequency of peers using a small test dose first was one of the strongest predictors of personal test dosing behavior (aOR = 1.51, 95% CI [1.34, 1.70]),

## Discussion

The three fatal overdose prevention behaviors were not strongly correlated, suggesting that harm reduction programs should provide people with a range of options for overdose prevention since different people may be able to enact different fatal overdose prevention behaviors (Yeo et al. 2022). Moreover, there were suboptimal levels of reported overdose prevention behaviors. Increasing these behaviors is a vital strategy to reduce fatal overdose risk. The findings of this study provide insights into the interplay of individual and social factors influencing overdose prevention behaviors among PWUO.

Across the three models examining the frequency of naloxone availability from others while using drugs, using a small test dose to assess potency, and using drugs alone, several consistent patterns emerged. One of the key findings of this study is the significant racial differences in overdose prevention behaviors. Black participants were more likely to use a small test dose compared to White participants. Prior research has suggested that historical distrust of healthcare institutions, systemic inequities in harm reduction service distribution, and variations in drug market dynamics may contribute to these differences (Gupta et al., 2014; Smith et al., 2025; Victor et al., 2024). Addressing these disparities requires culturally tailored interventions that improve naloxone availability in Black communities and potency testing among White PWUO.

Sex differences also emerged as a significant factor in overdose prevention behaviors. Female participants were more likely than males to use a small test dose and less likely to have naloxone available from others when using drugs. These findings align with previous research indicating that women who use drugs may face unique barriers to engaging in harm reduction or accessing harm reduction services such as naloxone distribution programs due to factors such as fear of stigma, caregiving responsibilities, or greater reliance on male partners for substance acquisition and safety (Mayock et al., 2015; Stewart et al., 2021). Tailored interventions for women who use drugs, including gender-specific harm reduction education and peer support networks, could help address these disparities and facilitate safer drug use practices.

Despite these similarities, several unique findings emerged across the models. Older participants were less likely to use with others who have naloxone, but age was not significantly associated with test dosing or using drugs alone. This finding suggests that naloxone-related behaviors may be associated with age, whereas other harm reduction strategies appear to be more stable across different age groups. The introduction of naloxone, a relatively new harm reduction intervention relative to test dosing or not using alone, may explain lower uptake of naloxone in older individuals who are also likely to have a drug use history dating to prior to naloxone availability. Interventions focused on providing information to enhance motivations and behaviors related to ensuring the availability and use of naloxone among older individuals may be needed. This is particularly relevant to cities like Baltimore, where older individuals are disproportionately impacted by overdose death.

Perceptions of being negatively judged for carrying Narcan were not significantly associated with two of the outcomes and positively associated with naloxone availability when using drugs with others, suggesting that this type of stigma surrounding naloxone does not appear to be a major determinant of these harm reduction behaviors. In contrast, the belief that carrying naloxone is helping the community was significantly associated with more frequent test-dosing. This highlights the importance of community-centered harm reduction messaging.

Another important finding was the role of social networks and peer influence in harm reduction behaviors. Having a running buddy was strongly associated with lower odds of using drugs alone, reinforcing the idea that social connections serve as a critical protective factor against solitary drug use, which is a risk factor for overdose death. Additionally, participants who perceived their peers as using test doses were more likely to adopt this behavior themselves, and this was also associated with lower odds of using drugs alone, underscoring the importance of social modeling in harm reduction. These findings highlight the significance of peer influence in harm reduction practices, suggesting that leveraging social networks can enhance the adoption of overdose prevention strategies (Bolinski et al., 2019; Green et al., 2009; Holloway et al., 2018; Latkin et al., 2004). Expanding peer-based naloxone distribution programs by integrating additional harm reduction training may be an effective approach for promoting safer drug use behaviors.

Our study also found that psychological factors, such as comfort with administering naloxone and perceived community benefits of carrying naloxone, were associated with higher engagement in harm reduction behaviors. Participants who were comfortable using naloxone on others were less likely to use drugs alone. These findings suggest that training and messaging on overdose prevention should focus on enhancing self-efficacy (Petrovitch et al., 2024).

Study limitations should be noted. The data were self-reported, and responses may have been influenced by social desirability and other biases. Future studies may want to assess overdose prevention behaviors during the last drug use episode to obtain a more precise assessment of these behaviors. Moreover, the study design was cross-sectional. Data was collected over 26 months, and street drug composition may have changed over this period. There was not a detailed social network inventory or a social norms scale administered, and we did not examine all possible overdose prevention behaviors. The community-based sampling method may have introduced biases.

Our findings underscore the need for multifaceted harm reduction interventions that consider the intersecting influences of race, gender, withdrawal symptoms, social networks, and behavioral factors. Addressing racial disparities in naloxone access requires targeted outreach and community-driven harm reduction initiatives (Bolinski et al., 2019). Gender-responsive interventions should provide tailored support for women who use drugs, addressing barriers such as stigma and caregiving responsibilities. Expanding peer-based harm reduction programs and leveraging social networks to normalize safer drug use behaviors could further enhance the effectiveness of overdose prevention strategies (Harris et al., 2020). Future research should explore the underlying reasons for racial and gender disparities in harm reduction engagement, as well as investigate how social network and peer based interventions can be optimized to maximize their impact. Additionally, qualitative research could provide deeper insights into the barriers to engaging in overdose prevention behaviors (Mercer et al., 2021).

## Conclusion

This study highlights the complex and multifaceted nature of overdose prevention behaviors among PWUO. By identifying key sociodemographic, social, and behavioral determinants of harm reduction engagement, these findings contribute to the evidence base needed to develop more effective interventions. Addressing structural barriers, enhancing peer-driven harm reduction strategies, and tailoring interventions to specific demographic groups will be essential in mitigating the opioid crisis and reducing overdose-related harm.

## Figures and Tables

**Table 1.** Demographic and overdose prevention behaviors (*N* = 783).

Demographic factors	Frequency	Percentage

**Race**		
Black	560	71.5
White	178	22.7
Other	45	5.8
**Sex Assigned at Birth**		
Male	479	61.2
Female	304	38.8
**Highest Level of Education**		
Grade 11 or less	230	29.4
Grade 12 or GED	353	45.1
Some college, Associates, or Technical Degree	178	22.7
Bachelor’s Degree or Greater	22	2.8
**Homeless in Past 6 Months**		
Yes	351	44.8
No	432	55.2
**Employment Status**		
Employed, full-time	28	3.6
Employed, part-time	49	6.3
Unemployed	337	43.0
Retired	32	4.1
Student	4	0.5
Disabled/unable to Work	333	42.5
**Overdose Prevention Behaviors**		
**Use a Small Test Dose Before Using**		
Always	328	41.9
Often/Sometimes	292	37.3
Rarely/Never	163	20.8
**Use Drugs Alone**		
Always/Often	376	48.0
Sometimes	227	29.0
Rarely/Never	180	23.0
**When you are using with others, how often do they have Narcan with them?**		
Always/Often	154	23.37%
Sometimes	234	35.51%
Rarely/Never	271	41.12%

**Table 2.** Social network, social norms, and comfort with overdose prevention (*N* = 783).

Social Network Variables	Frequency	Percentage

**Thinking of the people you know who use drugs, how many do you think have Narcan?**		
None	97	12.4
Some	449	57.3
Half	74	9.5
Most	120	15.3
All	43	5.5
**How often do you buy drugs from the same person?**		
Always	271	34.6
Often	387	49.4
Sometimes	97	12.4
Rarely	25	3.2
Never	3	0.4
**Do you have a running buddy?**		
Yes	477	60.9
No	306	39.1
**How often do people you use with try a small dose first?**		
Always	65	8.3
Often	105	13.4
Sometimes	201	25.7
Rarely	190	24.3
Never	222	28.4
**How comfortable are you using nasal Narcan?**		
Very comfortable	527	67.3
Somewhat comfortable	146	18.6
Somewhat uncomfortable	60	7.7
Very uncomfortable	50	6.4

**Table 3.** Attitudes and perceptions of overdose prevention behaviors (*N* = 783).

	If I carry Narcan, I am doing something that is helping the community.	If carry Narcan, I Feel Negatively Judged by people in my community	Sometimes when I feel sick or in withdrawal from not having my fix, I don’t think about overdose prevention
Response category	Frequency, (%)	Frequency, (%)	Frequency, (%)

Strongly Agree	409 (52.2%)	77 (9.8%)	223 (28.5%)
Agree	329 (42.0%)	137 (17.5%)	305 (39.0%)
Neither Agree nor Disagree	18 (2.3%)	124 (15.8%)	59 (7.5%)
Disagree	21 (2.7%)	334 (42.7%)	147 (18.8%)
Strongly Disagree	6 (0.8%)	111 (14.2%)	49 (6.3%)

**Table 4.** Ordered logistic regression model of frequency of using drugs alone (*N* = 783).

	Unadjusted Model	Adjusted Model
Independent variables	OR (95% CI)	aOR (95% CI)

Race (White vs. Black)	**0.64 (0.46–0.87)**	**0.57 (0.39–0.84)**
Race (Other vs. Black)	0.77 (0.43–1.37)	0.71 (0.39–1.30)
Age	1.00 (0.99–1.02)	1.00 (0.99–1.02)
Sex (Female vs. Male)	**0.76 (0.58–0.99)**	0.89 (0.67–1.19)
Education (Grade 12 or GED vs. <Grade 12)	1.08 (0.79–1.47)	1.23 (0.89–1.70)
Education (Some college, Associates vs. <Grade 12)	1.01 (0.70–1.45)	1.11 (0.76–1.62)
Education (Bachelor’s degree or greater vs. <Grade 12)	1.37 (0.59–3.16)	1.50 (0.64–3.55)
Number of people in drug network who have Narcan	0.89 (0.79–1.01)	0.93 (0.81 –1.05)
Frequency of buying drugs from the same person	1.10 (0.93–1.30)	--
Have a running buddy(ies)	**0.50 (0.38–0.66)**	**0.48 (0.36–0.64)**
Belief that carrying Narcan is doing something to help the community	1.01 (0.85–1.20)	--
Feel negatively judged by people if carry Narcan.	0.97 (0.87–1.08)	--
Withdrawal reducing overdose prevention consideration	0.99 (0.89–1.10)	--
Comfortable using nasal Narcan on someone else	**0.79 (0.67–0.92)**	**0.82 (0.70–0.97)**
Frequency of network members using a small dose first to test the strength of their drug.	**0.87 (0.78–0.97)**	**0.87 (0.78–0.97)**
Number of places used drugs in past 30 days	**1.05 (1.01–1.09)**	**1.08 (1.04–1.13)**

**Bold = *p* < 0.05**. (1) Rarely/Never, (2) Sometimes, (3) Always/Often

**Table 5.** Ordered logistic regression model of frequency of naloxone availability when using drugs (*N* = 659).

Independent variables	Unadjusted OR (95% CI)	Adjusted OR (95% CI)

Race (White vs. Black)	**1.58 (1.13–2.19)**	1.28 (0.85–1.92)
Race (Other vs. Black)	**2.05 (1.04–4.03)**	1.67 (0.82–3.42)
Age	**0.98 (0.96–0.99)**	**0.98 (0.96–0.99)**
Sex (Female vs. Male)	0.75 (0.56–1.01)	**0.72 (0.52–1.00)**
Education (Grade 12 or GED vs. <Grade 12)	1.19 (0.85–1.68)	1.04 (0.72–1.51)
Education (Some college, Associate’s vs. <Grade 12)	1.23 (0.83–1.81)	0.98 (0.65–1.50)
Education (Bachelor’s degree or greater vs. <Grade 12)	1.10 (0.47–2.56)	0.98 (0.38–2.47)
Number of people in drug network who have Narcan	**2.25 (1.93–2.63)**	**2.25 (1.91–2.64)**
Frequency of buying drugs from the same person	**1.29 (1.07–1.56)**	1.12 (0.91–1.39)
Have a running buddy	**1.88 (1.39–2.55)**	**1.74 (1.25–2.42)**
Belief that carrying Narcan is doing something to help the community	0.98 (0.81–1.19)	--
Feel negatively judged for carrying Narcan	1.10 (0.97–1.24)	**1.15 (1.01–1.31)**
Withdrawal reducing overdose prevention consideration	1.05 (0.94–1.19)	--
Comfortable using nasal Narcan on others	1.13 (0.96–1.34)	1.12 (0.93–1.35)
Frequency of network members using a test dose of drugs	**1.25 (1.11–1.41)**	**1.24 (1.09–1.40)**
Number of places used drugs in past 30 days	1.04 (0.99–1.08)	1.01 (0.96–1.06)

**Bold = *p* < 0.05**. (1) Rarely/Never, (2) Sometimes, (3) Always/Often

**Table 6.** Ordered logistic regression model of frequency of testing drugs for potency (*N* = 783).

Independent Variables	Unadjusted OR (95% CI)	Adjusted OR (95% CI)

Race (White vs. Black)	**0.45 (0.33–0.62)**	**0.54 (0.37–0.78)**
Race (Other vs. Black)	0.96 (0.55–1.68)	0.97 (0.54–1.75)
Age	**1.02 (1.01–1.03)**	1.01 (0.99–1.02)
Sex (Female vs. Male)	**1.36 (1.04–1.78)**	**1.42 (1.06–1.89)**
Education (Grade 12 or GED vs. <Grade 12)	0.91 (0.67–1.25)	0.91 (0.65–1.25)
Education (Some College, Associate’s vs. <Grade 12)	0.90 (0.62 –1.29)	0.87 (0.59–1.28)
Education (Bachelor’s degree or greater vs. <Grade 12)	0.90 (0.39–2.10)	0.89 (0.37–2.12)
Number of people in drug network who have Narcan	1.10 (0.97–1.25)	1.06 (0.93–1.21)
Frequency of buying drugs from the same person	**1.41 (1.19–1.67)**	**1.31 (1.09–1.57)**
Have a running buddy	1.15 (0.88–1.50)	--
Belief that carrying Narcan is doing something to help the community	**1.43 (1.18–1.72)**	**1.34 (1.11–1.63)**
Feel negatively judged for carrying Narcan	1.05 (0.94–1.17)	--
Experiencing withdrawal reducing overdose prevention consideration	**0.87 (0.78–0.97)**	0.90 (0.80–1.01)
Comfortable using nasal Narcan on others	**1.21 (1.04–1.40)**	**1.20 (1.02–1.40)**
Frequency of network members using a test dose of drugs	**1.53 (1.37–1.71)**	**1.51 (1.34–1.70)**
Number of places used drugs in past 30 days	**0.94 (0.90–0.97)**	0.97 (0.93–1.01)

**Bold = *p* < 0.05**. (1) Rarely/Never, (2) Sometimes/Often, (3) Always
